# Influence of family support on leisure activities and mental health in times of health crisis

**DOI:** 10.3389/fpsyg.2026.1736074

**Published:** 2026-01-29

**Authors:** Janeth Berenice García-Gallegos, Maritza Delgado-Herrada, Raquel Morquecho-Sánchez, Ignazio Leale, Jorge Zamarripa

**Affiliations:** 1 Universidad Autónoma de Occidente, Unidad Regional Guasave, Culiacan, Mexico; 2 Secretaría de Ciencia, Humanidades, Tecnología e Innovación (SECIHTI), Mexico City, Mexico; 3 Facultad de Organización Deportiva, Universidad Autónoma de Nuevo León, Nuevo Leon, Mexico; 4 Department of Psychology, Educational Sciences and Human Movement, Sport and Exercise Research Unit, Palermo, Italy

**Keywords:** COVID-19, family support, lockdown, Mexico, physical activity, sedentary behaviors

## Abstract

Physical activity is an essential pillar for promoting health and comprehensive well-being throughout life. During the COVID-19 pandemic, lockdown and social restriction measures significantly transformed family routines and lifestyles, leading to an increase in sedentary behavior and negative effects on mental health. This article aims to analyze the influence of family support on mental health, mediated by physical activity and sedentary behaviors during the COVID-19 lockdown. A total of 1,345 Mexican adults (68.1% women) participated, responding to validated instruments: the Social Support for Physical Activity Scale, the Godin-Shephard Leisure-Time Physical Activity Questionnaire, the Youth Leisure-Time Sedentary Behavior Questionnaire, and the Positive and Negative Affect Schedule (PANAS). Structural equation modeling analyses showed a satisfactory model fit (χ^2^/df = 6.71, CFI = 0.931, RMSEA = 0.060). Family support was positively associated with active leisure and, more weakly, with passive leisure. Furthermore, active leisure was related to greater positive affect, while passive leisure was associated with psychological distress. These results highlight the protective role of the family in promoting physical activity and emotional regulation in lockdown contexts. It is recommended to design family intervention programs that integrate educational, affective, and behavioral components to foster active lifestyles and strengthen resilience for future health crises.

## 1 Introduction

Physical activity is a fundamental pillar for health promotion at all stages of life. Regular practice significantly contributes to the prevention and control of chronic diseases such as cardiovascular, metabolic, and musculoskeletal conditions, as well as some types of cancer. It also yields psychological and cognitive benefits, including reduced depressive and anxiety symptoms, increased subjective well-being, and improved executive functions (Beauchamp and Eys, 2014; World Health Organization, 2022).

The COVID-19 pandemic caused a profound transformation in social and family dynamics, imposing conditions of lockdown and social contact restrictions that affected the physical and mental health of millions. This public health emergency altered the daily structure of families, lifestyle habits, and opportunities for physical activity, highlighting the importance of the family environment as an emotional and behavioral support network (Vélez-Álvarez et al., 2020; Ribot et al., 2020). Families, in particular, became spaces where behaviors related to active or passive leisure were modeled and reinforced, directly influencing patterns of well-being and mental health.

Several studies concur that the lockdown and the suspension of in-person activities led to an increase in sedentary behavior, a decline in emotional well-being, and a reduction in healthy habits, especially among children, adolescents, and young adults (del Paricio Castillo and Pando Velasco, 2020). These conditions were associated with a rise in anxiety and depressive symptoms, difficulties in coexistence, and greater exposure to psychosocial risk factors such as domestic violence or economic uncertainty (Cao et al., 2020; Crayne, 2020).

In this context, family support emerges as a specific and decisive component within social support, the latter interpreted as the perception and availability of emotional, instrumental, or informational assistance from various sources (Grey et al., 2020). Unlike general social support, family support comes exclusively from household members and close family, which gives it distinctive characteristics such as stable emotional bonds, shared responsibilities, and a daily influence on health habits (Hailey et al., 2023). Some studies have shown that this type of support constitutes a key resource for sustaining physical activity and reducing sedentary behaviors during confinement situations, by providing motivation, companionship, and a conducive environment for the adoption of healthy behaviors (Guo et al., 2022; Hailey et al., 2023). Its relevance also expands to the field of mental health, as family support has been shown to reduce perceived stress, moderate negative emotions, and promote psychological well-being in crisis contexts, such as those caused by the COVID-19 pandemic.

Furthermore, perceived social support has demonstrated a protective effect on mental health during the pandemic. The presence of a support network can reduce stress perception and improve sleep quality, factors that are closely related to mental health (Grey et al., 2020). For instance, a study by Grey et al. (2020) found that perceived social support was inversely related to levels of depression and sleep problems during the pandemic.

The relationship between social support and physical activity has also been explored in various contexts. A study by Hailey et al. (2021) indicated that perceived social support was positively associated with sustained physical activity during the lockdown. Similarly, Guo et al. (2022) found that social support was associated with a reduction in negative emotions among Chinese adolescents during the Omicron-related lockdown.

This article aims to analyze the influence of family support on mental health, mediated by physical activity and sedentary behaviors during the COVID-19 lockdown. Through a review of recent studies, it seeks to provide a comprehensive view of how the family environment can act as a determining factor in promoting healthy habits and mitigating the negative effects of confinement.

## 2 Method

### 2.1 Study design

This empirical study employed an associative, cross-sectional design with an explanatory purpose, using latent variables (Ato et al., 2013).

### 2.2 Population and sample

The target population consisted of Mexican adults over 18 years of age, residing in Guasave, Sinaloa. The eligibility criteria included having Mexican nationality, residing in Mexico, voluntarily agreeing to participate in the research, and being in confinement at the time of answering the questionnaire. Given the restrictive conditions derived from the COVID-19 pandemic, a non-probabilistic convenience sampling method was used (Hernández-Sampieri et al., 2014).

The sample size was estimated *a priori* using a calculator for structural equation models (Soper, 2021), which recommended a minimum of 689 participants for a model with five latent variables and 31 observed variables, considering a statistical power level of 0.95, an anticipated effect size of d = 0.20, and a significance level of 0.01. Ultimately, data were collected from 1,345 participants.

The final sample consisted of 68.10% women and 31.90% men, with a median age of 22 years and a mean age of 27.36 ± 11.31 years (range: 18–77 years). Most participants were students (58.88%), single (74.28%), and had undergraduate studies (68.10%), perceiving themselves as having a middle socioeconomic status (59.85%).

### 2.3 Instruments

#### 2.3.1 Social support for physical activity scale

To measure family support, the Social Support for Physical Activity Scale by Marcus and Forsyth (2009) was used. Participants indicated how often their family had said or done the things described in the items over the past 3 months on a Likert-type scale from 1 (none) to 5 (very often). An option “0 (Does not apply)” was included for cases where the statement was not applicable. An example item is “Did physical activity with me.” Following the recommendations of Marcus and Forsyth (2009), responses to items 7 and 8 were reverse-scored (1 = 5, 2 = 4, 3 = 3, 4 = 2, 5 = 1). Higher scores indicate greater social support.

#### 2.3.2 Godin-Shephard leisure-time physical activity questionnaire (LTPAQ)

To measure active leisure, the LTPAQ was used (LTPAQ; Godin, 2011; Godin and Shephard, 1985). The level of leisure-time physical activity was calculated based on recommendations by Godin (2011). First, the weekly frequencies of strenuous, moderate, and light activities were multiplied by nine, five, and three, respectively; these values correspond to energy expenditure categories measured in METs from a list of activities. The total weekly leisure-time physical activity score was then calculated in arbitrary units by summing the products of the separate components, as shown: Weekly leisure-time physical activity score = 9*(Vigorous) + 5*(Moderate) + 3*(Light). The instrument has shown adequate correlation between subjective and objective data and correctly classifies 69% of individuals as fit or unfit (Godin and Shephard, 1985), as well as healthy adults into active and insufficiently active categories (Amireault and Godin, 2015).

#### 2.3.3 Youth leisure-time sedentary behavior questionnaire (YLSBQ)

To measure passive leisure, the Youth Leisure-time Sedentary Behavior Questionnaire (YLSBQ; Cabanas-Sánchez et al., 2018) was used to quantify the time young people spend on a wide range of sedentary leisure-time behaviors. For this study, participants were asked to think about a typical week, before and during the lockdown, regarding the average estimated time dedicated to each behavior on weekdays (Monday to Friday) and weekends (Saturday and Sunday) separately. The average time per day for each behavior and composite category was calculated as follows: [(weekday time * 5) + (weekend time * 2)]/7. A total sedentary time score was obtained by summing the reported time for the 12 sedentary behaviors. The instrument has shown adequate reliability and temporal stability in recent studies (Cabanas-Sánchez et al., 2018).

#### 2.3.4 Positive and negative affect schedule (PANAS)

As an indicator of mental health, the PANAS (Watson et al., 1988) was used. This instrument consists of 20 adjectives, 10 of which measure positive emotions and the other 10 measure negative emotions. Responses are given on a 5-point Likert-type scale from 1 (never) to 5 (always). Higher scores indicate greater positive and negative affect, respectively.

### 2.4 Procedure

The study was conducted in accordance with official ethical guidelines, specifically the Mexican Official Standard NOM-012-SSA3-2012 and the Regulations of the General Health Law regarding Health Research (Secretaría de Salud, 1987). The research was approved by the Bioethics Committee of the Universidad Autónoma de Occidente, with official letter number CM-UAdeO 07.10/2020.

Data collection was performed virtually via a link to a questionnaire hosted on the QuestionPro® platform. The link was distributed through social networks such as Facebook®, Twitter®, and WhatsApp® during April, May, and June 2020. Before starting the questionnaire, participants received clear information about the study’s objectives, assurance that their questions would be answered, the option to withdraw at any time, and confidentiality of their data and identity. The privacy of all participants was protected (Article 16), and it was reiterated that the information obtained would be used exclusively for scientific purposes, maintaining anonymity and confidentiality (Article 17, Section I).

The data collection process included a prior explanation and virtual support from the surveyor, who remained available to resolve doubts and reiterate the voluntary nature of participation. Only those who provided informed consent could access the questionnaire.

### 2.5 Data processing and analysis

Descriptive statistics, reliability analyses of the scales using Cronbach’s Alpha and McDonald’s Omega coefficients, and correlation analyses were performed using SPSS Statistics V.22.0. Additionally, Confirmatory Factor Analyses (CFA) and a Structural Equation Model (SEM) were conducted with JASP software (version 0.18.3) (JASP Team, 2024) to analyze the effects of family support on active and passive leisure, and the influence of these on the positive and negative affect reported by participants (Hair et al., 2009; Cabanas-Sánchez et al., 2018; Soper, 2021).

Cronbach’s Alpha and McDonald’s Omega values of 0.70 or higher are considered acceptable (Hair et al., 2009). The adequacy of the data in the CFA and SEM was analyzed using different fit indices such as CFI, NNFI, RMSEA, and SRMS with the Diagonal Weighted Least Squares (DWLS) method. CFI and NNFI values greater than or equal to 0.95 indicate an acceptable fit (Hu and Bentler, 1999). For RMSEA, values less than or equal to 0.08 are considered satisfactory (Cole and Maxwell, 1985).

## 3 Results

### 3.1 Psychometric properties of the instruments

The reliability analyses revealed good internal consistency for all scales used, with Cronbach’s Alpha and McDonald’s Omega values ranging from 0.76 to 0.92 and 0.93, respectively, for the family support scale. All fit indices for the CFAs were acceptable to satisfactory (NNFI, CFI > 0.90; and RMSEA, SRMR < 0.08) (See Table 1).

**Table 1:** Psychometric properties of the instruments: internal consistency and confirmatory factor analysis.

Variable	*α*	*ω*	*χ*^2^	df	NNFI	CFI	RMSEA	SRMR
Family support	0.92	0.93	453.696	65	0.977	0.981	0.061	0.073
Sedentary behavior	0.76	0.76	448.542	51	0.905	0.927	0.070	0.063
Positive affect	0.91	0.91	905.697	103	0.972	0.976	0.070	0.069
Negative affect	0.92	0.92	905.697	103	0.972	0.976	0.070	0.069

*α*, Cronbach’s alpha; *ω*, composite reliability; *χ*^2^, Chi-square; df, degrees of freedom; NNFI, non-normed fit index; CFI, comparative fit index; RMSEA, root mean square error of approximation; SRMR, standardized root mean square residual.

### 3.2 Descriptive analysis and data normality

Descriptive data showed that the item with the highest mean on the family support scale was “Criticized or made fun of me for exercising” (reverse-scored item), while the item with the lowest mean was “Rewarded me for exercising.” Regarding physical activity, the majority of participants (69.1%) indicated they never performed any type of light, moderate, or vigorous exercise per week. For sedentary behaviors, the behavior with the highest score was “Talking on the phone or sending messages on WhatsApp,” and the one with the lowest score was “Playing on the computer/video games.” All skewness and kurtosis values were within ±1, indicating that the data follow a normal distribution (See Table 2).

**Table 2:** Descriptive results and Pearson correlation analysis between the study variables.

Variable	*M*	*SD*	Min	Max	*S*	*K*	1	2	3	4
1. Family support	2.857	0.638	1.23	5.00	0.083	−0.651	–			
2. Phyical activity	76.943	44.295	17	187	0.503	−0.651	0.338***	–		
3. Sedentary behavior	234.505	59.492	63.43	443.71	0.275	0.039	0.092***	0.205***	–	
4. Positive affect	4.284	1.345	1.00	7.00	−0.052	−0.589	0.192***	0.185***	0.090***	–
5. Negative affect	3.829	1.483	1.00	7.00	−0.073	−0.765	−0.009	0.057*	0.214***	−0.405***

M, mean; SD, standard deviation; Min, minimum; Max, maximum; S, skewness; K, kurtosis; *p < 0.05; **p < 0.01; ***p < 0.001.

### 3.3 Correlation analysis

Family support correlated positively with the average hours of physical activity and sedentary behaviors, as well as with positive affect. The average physical activity correlated positively with sedentary behaviors and with both positive and negative affect. Sedentary behaviors correlated positively with both positive and negative affect, and the correlation between the affects was negative and significant (See Table 2).

### 3.4 Structural equation model

**Figure 1:**
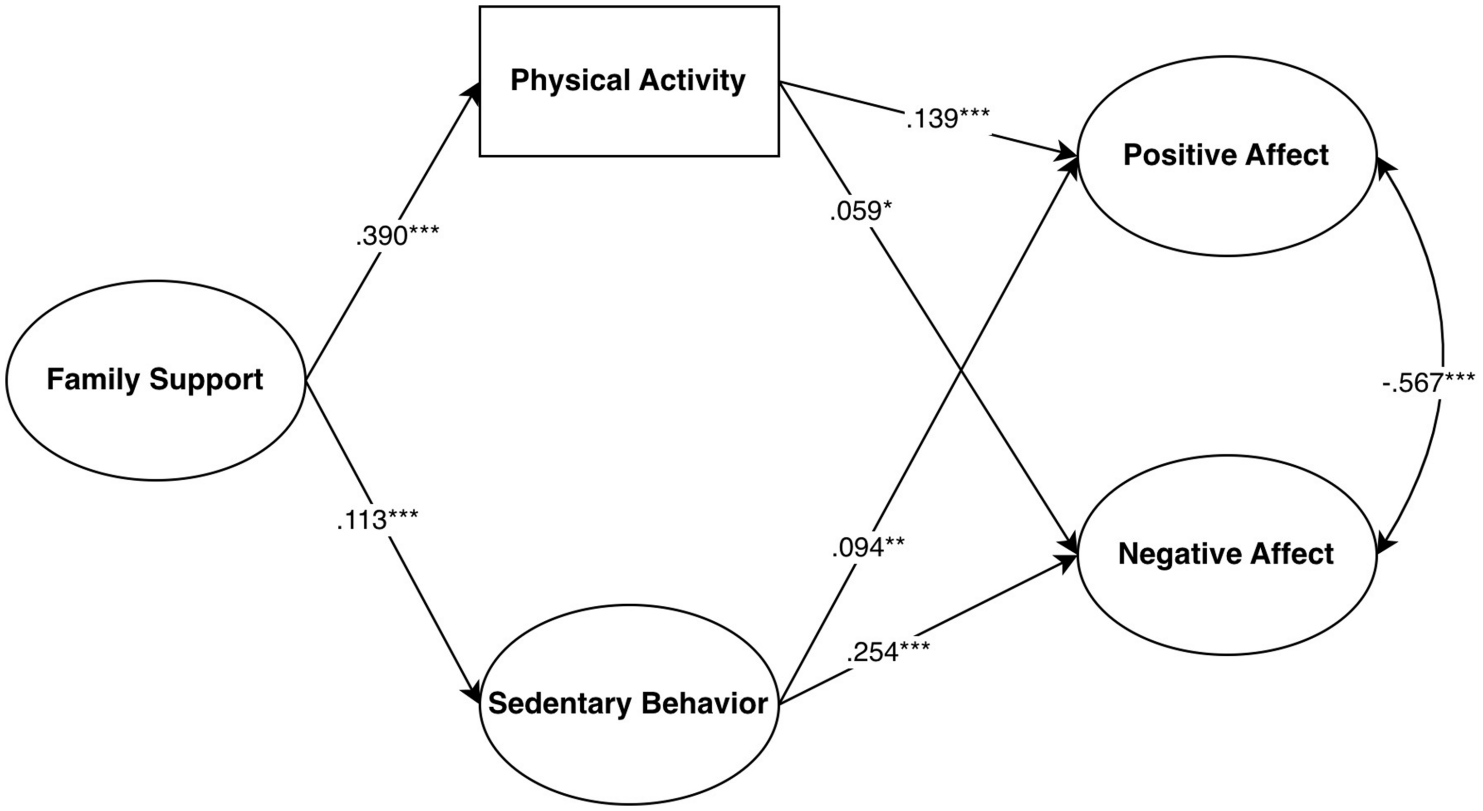
Standardized solution of the structural equation model of the relationships between family support, leisure types, well-being, and distress.

A structural equation model was developed to analyze the influence of family support on mental health, mediated by physical activity and sedentary behaviors. The model showed satisfactory fit indices (χ^2^ = 5441.07, *df* = 811, *p* < 0.001, χ^2^/df = 6.71, NNFI = 0.927, CFI = 0.931, RMSEA = 0.060, SRMR = 0.066). The relationship between family support and active leisure was stronger than its relationship with passive leisure. Active leisure was positively associated with well-being (positive affect) and slightly with distress (negative affect). In contrast, passive leisure showed a weaker relationship with well-being and a stronger one with psychological distress (See Figure 1).

In addition to the direct effects mentioned in the model, there were also indirect effects of family support on well-being through active leisure (*β* = 0.172, *p* < 0.001) and of family support on distress through passive leisure (*β* = −0.048, *p* < 0.001).

## 4 Discussion

The results of this study confirm the relevance of family support as a factor associated with the promotion of physical activity and mitigating the adverse effects of sedentary behavior and confinement on mental health. This relationship highlights the essential role of the family as a facilitator of overall well-being, influencing both active behaviors and emotional coping strategies developed during situations of social isolation (Farhang et al., 2024; Hailey et al., 2023; Guo et al., 2022).

The positive association between family support and active leisure indicates that families encouraging physical activity help their members maintain healthy exercise levels. This finding aligns with research suggesting that emotional support, shared motivation, and health-oriented family norms strengthen adherence to regular physical practice, self-confidence, and perceived self-efficacy (Bandura, 1989; Beauchamp and Eys, 2014). Such processes are particularly relevant in crisis contexts, where the family acts as a support system and promoter of healthy habits in the face of uncertainty (Cavicchioli et al., 2021; Fang et al., 2020).

Similarly, the positive correlation between active leisure and positive affect underscores the empirical evidence on the role of physical exercise in emotional regulation and the improvement of subjective well-being. Scientific literature has shown that frequent participation in physical activities increases endorphin release, improves sleep quality, and enhances interpersonal relationships—key elements for mental health (Hu et al., 2022; Jurak et al., 2020). Conversely, the prevalence of sedentary behaviors can decrease the perception of well-being and increase vulnerability to stress and anxiety, especially in contexts of prolonged social isolation (Stockwell et al., 2021; Cheval et al., 2021; Garcidueñas-Gallegos et al., 2023).

From an ecological and systemic perspective, the results affirm that the family environment acts as a microsystem of direct influence on health habits, validating the assumptions of Bronfenbrenner (1987) and Bandura (1989). The family not only provides material and emotional resources but also shapes behaviors and values oriented toward adopting active lifestyles. This learning process, through observation and positive reinforcement, fosters the re-establishment of a family culture of movement, even under conditions of spatial or social restriction.

From this perspective, health promotion interventions should explore comprehensive strategies that include different household members, combining educational, affective, and behavioral components. Designing programs that promote family physical activities, both in-person and virtual, can foster resilience, cohesion, and psychological well-being in future crisis or lockdown situations (Cavicchioli et al., 2021; World Health Organization, 2020).

## 5 Conclusion

In sum, the results of this study emphasize the need to re-evaluate the role of the family as an active agent in promoting healthy leisure and mental health. Fostering environments that balance active leisure with moments of rest and emotional connection can become a protective tool against stress, isolation, and the deterioration of well-being. Future research should employ longitudinal designs and mixed-methods approaches to analyze the causal mechanisms of family support on active behavior, as well as the differential impact across types of leisure and age groups. Additionally, incorporating neuropsychological measures and biomarkers could provide a deeper understanding of the physiological processes mediating this relationship.

This study stands out for its large and well-justified sample size, the use of validated instruments with good psychometric properties, and the application of structural equation modeling, which strengthens the robustness of its conclusions about the role of family support in physical activity and mental health during the COVID-19 lockdown. However, its cross-sectional design, non-probabilistic sampling in a single city, and the overrepresentation of young students, together with the exclusive use of self-report measures, limit causal inference and restrict the generalizability of the findings.

## Data Availability

The raw data supporting the conclusions of this article will be made available by the authors, without undue reservation.
